# Flow cytometric analysis of platelets type 2 diabetes mellitus reveals ‘angry’ platelets

**DOI:** 10.1186/s12933-016-0373-x

**Published:** 2016-03-31

**Authors:** Prashilla Soma, Albe Carina Swanepoel, Jeanette Noel du Plooy, Thandi Mqoco, Etheresia Pretorius

**Affiliations:** Department of Physiology, School of Medicine, Faculty of Health Sciences, University of Pretoria, Private Bag x323, Arcadia, Pretoria, 0007 South Africa

## Abstract

**Background:**

The function of platelets have extended way beyond the horizon of haemostasis and thrombosis, and are recognised as active participants in vascular inflammation, as well as in prothrombotic complications of cardiovascular diseases. We describe and compare platelet function in type II diabetes (with and without cardiovascular manifestation) and healthy individuals using scanning electron microscopy and flow cytometry.

**Methods:**

Thirty subjects were recruited per group and informed consent was obtained from all participants. Diabetic patients were recruited from the diabetic clinic of the Steve Biko Academic Hospital (South Africa). Blood samples were drawn from all participants so that platelet specific antigens were analyzed in citrated whole blood. The platelet parameters used in the study were platelet identifiers (CD41 and CD42) and markers of platelet activation (CD62 and CD63).

**Results:**

Results show that, compared to healthy individuals, both diabetic groups showed a significant difference in both platelet identifiers (CD41-PE, CD42b-PE) as well as markers indicating platelet activation (CD62P-PE and CD63-PE).

**Interpretation:**

The flow cytometric data shows that the platelet surface receptors and platelet activation are statistically elevated. This is suggestive of enhanced platelet activation and it appears as if platelets are displaying ‘angry’ behaviour. The lysosomal granules may play a significant role in diabetes with cardiovascular complications. These results were confirmed by ultrastructural analysis.

## Background

It is plausible to underestimate the impact of platelets in clinical medicine, when one considers that these blood cells are only 1.5–3 μm in size, survive for approximately 8–10 days, and are mere fragments of megakaryocyte cytoplasm [1–3]. The function of platelets have extended way beyond the horizon of haemostasis and thrombosis. In fact, they are now recognised as active participants in initiating and sustaining vascular inflammation as well as in prothrombotic complications of cardiovascular diseases [1]. Platelets have been assigned multiple attributes and have been described in inflammatory conditions such as atherosclerosis, arthritis and tumour metastasis [1]. Due to the multifunctional role of platelets, they are an accessible and important inflammatory marker for disease pathophysiology [4, 5]. Platelets are activated when they are in contact with damaged vascular endothelium [5], and once activated, they are able to secrete a wide spectrum of inflammatory mediators that exert both local and systemic effects [6].

Platelet activation is also the mechanism implicated in the pathogenesis of chronic medical conditions such as atherosclerosis, coronary vascular disease and cerebrovascular disease [3]. Due to inflammation there is an imbalance between procoagulant and anticoagulant properties of the endothelium with subsequent local stimulation of the coagulation cascade [7]. Another feature of inflammation is a multitude of interactions between leukocytes, endothelial cells and platelets. More importantly, regardless of its aetiology, inflammation causes endothelial activation [7]. In diabetes mellitus, endothelial dysfunction is one of the mechanism ascribed to increased atherothrombotic risk [8]. With cigarette smoking, the endothelium becomes activated and induces the intrinsic coagulation pathway. This results in platelet activation and enhanced platelet aggregation, which in turn causes thrombin stimulation and fibrin formation [9]. Abnormal platelet activation, platelet count and volume have been implicated as risk factors of ischaemic stroke [10].

Once platelets are activated, they initiate reactions whereby changes in the level of expression of surface glycoproteins (GP) results, which act as receptors for platelet agonists and for adhesive proteins, involved in platelet aggregation. Platelet activity can be measured using various fluorescently labelled markers in flow cytometry. As flow cytometry allows the simultaneous detection of surface antigens in a sensitive and specific manner, it is therefore possible to examine aspects of the platelet membrane activity—see Table 1 for examples of available platelet markers.

**Table 1:** Platelet parameters measured in this study

Cluster of differentiation	Glycoprotein (GP)	Function
CD41	GPIIb	Anti-CD41a reacts with platelet membrane GPIIb in the intact complex with GPIIIa but not with GPIIb or GPIIIa separately. Useful in the identification and enumeration of platelets
CD42	GPIb	Anti-CD42b reacts with platelet membrane GPIb
CD62	P-Selectin	Anti-CD62 reacts with α-granule membrane protein which is expressed on the surface of activated platelet
CD63	GPIV	Anti-CD63 reacts with lysosomal granule-membrane glycoprotein that is expressed on surface of activated platelet

In this study CD41-FITC (fluorescein isothiocyanate) and CD41, Cd42b, CD62, CD63-PE (phycoerythrin) was used

### Platelets in inflammatory conditions

There is a strong indication that platelets also have relevant functions in inflammation [11]. In fact, it was shown that thrombosis and inflammation share many key molecular mechanisms and that they are fundamentally linked processes [7]. It is now recognised that vascular inflammation is the key underlying mechanism in atherogenesis and atheroprogression. The evidence of platelets being fundamental mediators in the initiation and maintenance of a chronic proinflammatory milieu is provided by the direct interactions with inflammatory cells and secretion of autocrine and paracrine effector molecules [12]. Another emerging concept is the significant role of platelet-mediated recruitment of leucocytes in the propagation, progression and pathogenesis of atherosclerotic disease. Platelets can interact with leucocytes: (a) during haemostasis, when there is vascular damage and recruit leucocytes to the growing thrombus, (b) when endothelial cells are stimulated thereby adhering and activating platelets and then bridge blood-borne leucocytes to the vessel wall and (c) in the formation of heterotypic aggregates prior to contact with endothelial cells when adhesion between platelets and leucocytes occur in the blood [13].

It is well known that in subjects with type 2 diabetes mellitus, function of platelets is impaired. In fact, a sub-threshold stimuli is needed to activate platelets which are constantly in activation despite the lack of a major plaque event and have thus been defined as ‘angry platelets’ [14]. This is significant as it has been postulated that circulating platelets in subjects with untreated type 2 diabetes mellitus are in a hyperactive state and are implicated as etiologic factors in thrombotic complications [15] which are accelerated in diabetics [16]. Of note is the finding of hyperactive platelets in metabolically controlled diabetics without cardiovascular complications [17]. In addition, expression of P-selectin is increased on the surface of platelets in patients presenting with symptomatic coronary artery disease, making it a marker of ‘angry platelets’ [18].

Diabetes with cardiovascular complication may also lead to acute conditions like thrombo-embolic ischemic stroke. Multiple studies regarding the activity of platelets in acute stroke have been performed. Results obtained from these studies (acute ischaemic stroke) showed increased mean platelet volume, platelet aggregation enhancement in post-ischaemic stroke, increased α-and dense granule release and statistically significant increase in expression of P-selectin (CD62P), CD63, and thrombospondin [19]. The study by Marquardt and co-workers investigated the time course of platelet activation after ischaemic stroke. They found a significant increase in CD62P and CD63 expression within 24 h post cerebral ischaemia. In addition, it was also shown that CD62P expression declines during the first weeks after stroke, whereas CD63 expression remains increased for at least 3 months after stroke [20].

Another confounding factor together with diabetes is cigarette smoking. Multiple studies provides evidence on the many adverse effects of smoking on the cardiovascular system. This includes: (a) it causes endothelial dysfunction [21, 22]; (b) increases inflammation [23]; (c) it alters the lipid profile and creates an atherogenic setting; (d) promotes atherosclerotic progression by enhancing oxidative stress, lipid peroxidation and mitochondrial damage [23, 24]; (e) it destabilizes atherosclerotic plaque by increasing matrix metalloproteinases [25]; (f) increases platelet activation and activates coagulation cascade with subsequent atherothrombosis [26, 27]. Flow cytometric findings in the research by Al-Dahr, showed a decrease in CD41b with an increase in CD40 and CD62 [28]. This paper, therefore investigates the functional role of platelets in diabetes, with and without cardiovascular involvement using flow cytometry and scanning electron microscopy to look at platelet ultrastructure.

## Methods

### Participants

Thirty healthy individuals were used as controls. These individuals were non-smokers, who did not use any chronic medication and did not have a history of thrombotic disease. Sixty diabetic subjects (type 2) were recruited from the Steve Biko Academic Hospital, diabetic clinic in South Africa. Inclusion criteria included: (a) subjects older than 18 years and willing to provide informed consent, (b) subjects with known diagnosis of diabetes, (c) for the cardiovascular group, history of previous myocardial infarction, peripheral arterial disease, stroke or coronary arterial bypass grafting. Exclusion criteria included: (a) subjects hemodynamically unstable and (b) subjected with documented life threatening disease (malignancy, HIV/AIDS). Two groups of thirty each were distinguished, with and without cardiovascular complications. Five millileters of blood was drawn into a citrate tube, from each participant. Ethical clearance was obtained for this study from the University of Pretoria Human Ethics Committee. Informed consent was obtained from all participants.

### Ultrastructural analysis

Scanning electron microscopy was used to prepare platelets from platelet rich plasma (PRP) according to previously described methods [29]. Platelets from individuals with diabetes, cerebrovascular disease and smoking were compared to platelets from healthy individuals.

### Flow cytometry

For each blood sample taken four tubes was prepared; each tube containing 1 ml sheath fluid from Beckmann and Coulter and 20 µl of blood. The various tubes were stained with 20 µl of CD41-FITC (fluorescein isothiocyanate) and 20 µl of one of the following probes: CD41-PE (phycoerythrin), CD42b-PE, CD62P-PE and CD63-PE (from Beckman Coulter). The samples stained with different probes, were incubated at room temperature in the dark for 20 min before being analyzed by a flow cytometer (FC 500, Beckman Coulter). The surface expression of platelet receptors was determined by flow cytometry using the different monoclonal antibodies as indicated in Table 1.

Forward scatter and 90º side scatter were displayed on logarithmic scales. Two platelet gates were set. The first gate was set according to the morphological characteristics of platelets while the second gate was set according to CD41-FITC fluorescence, a platelet specific marker. The fluorescence of the different antibodies was plotted on 256-channel log histograms. The results were expressed in arbitrary units as mean channel fluorescence intensity (MCFI).

### Statistical analysis

For each participant the MCFI was calculated as the mean fluorescence of a large sample of platelets (10,000 platelets per individual), the well-known Central Limit Theorem assures us that the Normal distribution is a close approximation for the distribution of the MCFIs for the experimental groups. GraphPad Prism 5 was employed to perform one-way ANOVA for all statistical analysis, with a p value of ≤0.005 considered significant. Post-hoc Dunnett’s Multiple Comparison Test was performed to compare the two diabetic groups to the controls.

## Results

**Fig. 1:**
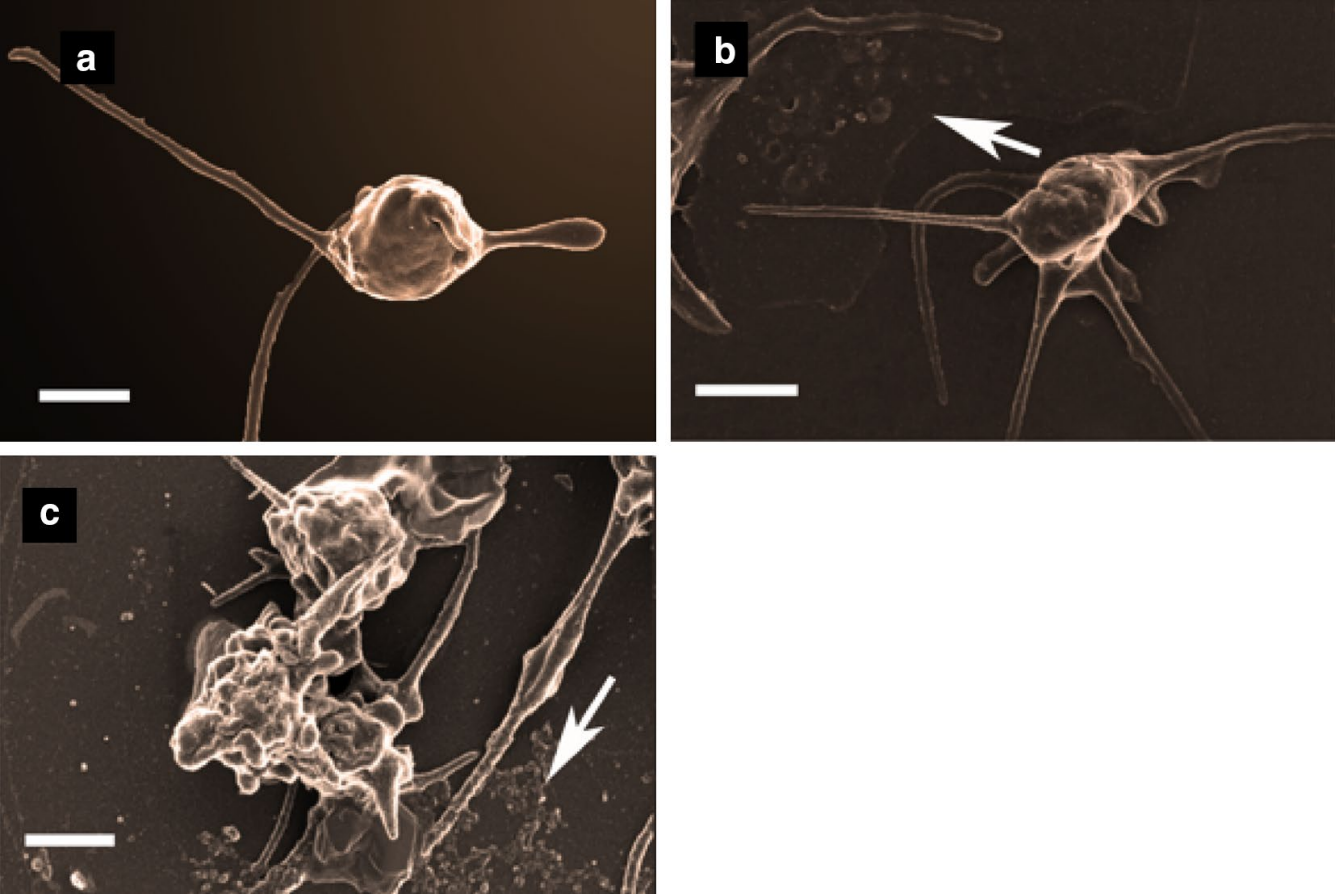
Micrographs showing representative platelets in (**a**) a typical platelet as seen in a healthy individual where platelets are *disc-shaped* with a few pseudopodia formation due to contact activation; **b** Type II diabetes without CVD; *arrow* shows platelet spreading and pseudopodia formation; **c** Platelets from type II diabetes with CVD now showing extensive spreading and microparticle formation (*arrow*). *Scale bar* 1 μm

Table 2 shows the demographic data of our study population. SEM analysis of the platelets from the three groups showed that there is a progressive change in platelet structure between the groups. Representative micrographs of the ultrastructure of platelets from healthy individuals, and individuals with diabetes (with and without cardiovascular manifestations), are shown in Fig. 1. Healthy platelets prepared for SEM, typically show slight contact activation, where minimal pseudopodia formation is visible (Fig. 1a). However, during inflammation, platelets form numerous pseudopodia, with microparticle formation, as well as spreading and extensive clumping, which is the hallmark of over-, or hyperactivation. This hyperactivation is seen in platelets from individuals with diabetes with and without CVD (Fig. 1b, c). However, diabetic patients with CVD are characterised by an increased presence of hyperactivation and microparticle formation Fig. 1c. Following the ultrastructural analysis we performed flow cytometry on the control and two diabetic groups. We found that the ultrastructural results were fully supported by the flow cytometry results discussed below.

**Table 2:** Baseline demographic data and clinical characteristics of the study population

Variable	Controls (n = 30)	Diabetics without CVD (n = 30)	Diabetics with CVD (n = 30)
Age, years	25 ± 9.64	53 ± 13.7	61 ± 9.4
Males, n (%)	6 (20)	11 (37)	20 (67)
Females, n (%)	24 (80)	19 (63)	10 (33)
Hypertension, n (%)		17 (57)	26 (87)
Diabetic treatment			
Insulin, n (%)		5 (17)	3 (10)
Oral agents only, n (%)		16 (53)	12 (40)
Oral and insulin, n (%)		9 (30)	15 (50)
HBA1c %^a^		9.0 ± 2.6	8.5 ± 1.7
Cardiovascular complications			
Previous MI, n (%)			19 (63)
PAD, n (%)			2 (7)
CABG, n (%)			9 (30)
Essential medication			
ACEI, n (%)		10 (33)	20 (67)
Ca-antagonist, n (%)		8 (27)	3 (10)
Β-blocker, n (%)		3 (10)	11 (37)
Nitrates, n (%)		1 (3)	14 (47)
Statins, n (%)		10 (33)	25 (83)
Disprin, n (%)		6 (20)	20 (67)
Warfarin, n (%)		2 (7)	5 (17)

Data expressed as mean ± (SD) or n (%)

*MI* myocardial infarction, PAD peripheral arterial disease, *CABG* coronary arterial bypass grafting, *ACEI* angiotensin converting enzyme inhibitor, *Ca*-*antagonist* calcium antagonist, *HBA1c* haemoglobin A1c

^a^Not all 60 subjects had this test completed (results are available for 50 % of the subjects)

CD41-PE and CD42b-PE MCFI were significantly elevated in both diabetic groups when compared to healthy individuals (p value <0.001). CD62P-PE and CD63-PE MCFI were significantly decreased for both diabetic groups. The percentage activated platelets indicated with CD62P-PE and CD63-PE were significantly increased in both the diabetic groups. It should be noted that the platelet activation indicated CD63-PE showed the diabetic group with cardiovascular complication to have the largest percentage of activated platelets.

## Discussion

### Diabetes

Increased expression of platelet activation markers CD31, CD36, CD49b, CD62P and CD63 was confirmed by Eibl and co-workers when type 2 diabetics were compared with normal individuals [30]. In fact, increased expression of CD63 and CD62, enhanced platelet activation, and aggregation are viewed as one of the major causes of atherosclerosis and thrombosis in diabetes [31]. CD62P is found in the α-granules of platelets and are used as markers for activated platelets, while their absence suggests a resting state [32]. A feature that appears strongly in diabetics is that of platelet hyperaggregation. This is prevalent in both type 1 and type 2 diabetics [33]. From a pathophysiological view, this is significant as hyper-aggregated platelets have a tendency to block blood vessels [5], contributing to atherothrombotic complications in diabetics. CD63 is a 53 kDa lysosomal membrane protein identified on surface of activated platelets after release reaction [34, 35].

Prevention of early platelet adhesion to the damaged vessel wall by blocking platelet surface receptors GPIbα or GPVI protects from stroke without provoking bleeding complications. In addition, downstream signalling of GPIbα and GPVI has a key role in platelet calcium homeostasis and activation [36]. The CD42b MoAb used in this research, specifically binds to the platelet GPIbα. GPIbα forms part of the GPIb-IX-V complex which is the receptor for von Willebrand’s factor and is known as von Willebrand’s factor-dependant adhesion receptor. According to De Meyer and co-workers in 2011, the importance of GPIbα far exceeds that of VWF in arterial thrombosis and GPIbα is a central receptor in different vascular processes of thrombosis and inflammation, all of which may contribute to the progression of ischemic stroke [37]. Furthermore, engagement of GPIb-IX-V by von Willebrand factor (VWF) mediates platelet adhesion to damaged vessels and triggers platelet activation and thrombus formation in heart attacks and stroke [38].

Flow cytometric analysis found a significant increase in platelet activation regarding CD62 (P-selectin) for the diabetics group while the CD62 MCFI values decreased compared to the controls as shown in Table 3. This unexpected finding may be attributed to the fact that P-selectin can be cleaved from the membrane surface after activation releasing P-selectin into the plasma known as soluble P-selectin (sP-selectin). The exact mechanism of this shedding is unknown but several mechanisms have been suggested including cleavage by serum proteases or non-specific enzymes or by simple shedding [39]. However, studies have shown an increase in both P-selectin on the surface of platelets and sP-selectin indicating that diabetes with or without cardiovascular complications are associated with chronic activation of platelets as P-selectin is being shed from activated platelets and as new P-selectin is being expressed on recently activated platelet [40]. This study finding echoes results found by Véricel et al. [17] whom also discovered hyperactive platelets in metabolically controlled diabetics without cardiovascular complications. The diabetic subjects with cardiovascular disease, recruited in this study were those with ischaemic events many months and years prior to recruitment into this study. Our finding is in keeping with persistently hyperactivated platelets.

**Table 3:** Analysis of control and diabetic groups (with or without CVD), results presented as mean ± standard deviation (SD) of MCFI and percentage activated platelets

MoAb	Controls (n = 30)	Diabetes without CVD^s^ (n = 30)	Diabetes with CVD (n = 30)
CD41-PE	21.08 ± 8.74	40.18 ± 14.56*	47.81 ± 24.49*
CD42b-PE	14.43 ± 1.84	24.78 ± 11.98*	24.09 ± 7.27*
CD62P-PE X-mean	30.86 ± 11.37	17.96 ± 1.95*	17.79 ± 2.266*
CD62P-PE % activated platelets	71.01 ± 16.20	92.65 ± 3.67*	92.11 ± 4.31*
CD63-PE X-mean	16.40 ± 3.32	13.05 ± 3.68**	13.38 ± 3.12**
CD63-PE % activated platelets	37.84 ± 17.59	54.39 ± 32.92**	61.24 ± 29.73**

n = 10,000 platelets total analysed for each participant of each group

Statistically significant differences: * p < 0.001, ** p < 0.005

**Fig. 2:**
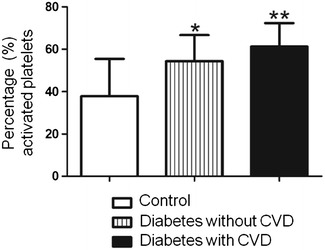
Percentage activated platelets identified with CD63-PE. The *asterisk* represent the q-value as calculated with the Newman–Keuls multiple comparison test: *3.291; **6.653. The q-value represents the studentized range value

The CD63 MoAb recognizes the activation-specific fusion of the lysosomal granule membrane with the plasma membrane, therefore it only binds on the surface of activated platelets and is a useful tool to use in the identification of activated platelets [41]. In our study CD63 percentage activated platelets were significantly increased compared to the healthy controls. The diabetic group with cardiovascular complication showed the greatest percentage of platelet activation (61.24 % activation) while the diabetic group without cardiovascular complications showed a slightly lower activation percentage (54.39 % activation), as shown in Fig. 2. It appears as if CVD may play a role in platelet hyperactivation with lysosomal involvement.

## Conclusion

This study adds to the body of evidence that diabetic patients have ‘angry platelets’. Platelet membrane markers and percentage activated platelets were increased in diabetics with and without CVD. These results support and confirm the numerous papers suggesting that platelets play an important and possibly key role in the inflammatory profile of diabetic and cardiovascular patients and their health should play a key role in a patient-orientated precision medicine approach.
